# Dose‐dependent effect of impaired renal function on all‐cause mortality in patients following percutaneous coronary intervention

**DOI:** 10.1002/clc.23877

**Published:** 2022-06-27

**Authors:** Thosaphol Limpijankit, Mann Chandavimol, Suphot Srimahachota, Anek Kanoksilp, Poj Jianmongkol, Sukanya Siriyotha, Ammarin Thakkinstian, Wacin Buddhari, Nakarin Sansanayudh

**Affiliations:** ^1^ Department of Medicine, Division of Cardiology, Faculty of Medicine, Ramathibodi Hospital Mahidol University Bangkok Thailand; ^2^ Department of Medicine, Division of Cardiovascular Diseases King Chulalongkorn Memorial Hospital Bangkok Thailand; ^3^ Department of Cardiology Central Chest Institute of Thailand Nonthaburi Thailand; ^4^ Department of Cardiology Buddhachinaraj Hospital Phitsanulok Thailand; ^5^ Department of Clinical Epidemiology and Biostatistics, Faculty of Medicine, Ramathibodi Hospital Mahidol University Bangkok Thailand; ^6^ Department of Internal Medicine, Cardiology Unit Pharmongkutklao Hospital Bangkok Thailand

**Keywords:** all‐cause mortality, chronic renal failure, Impaired renal function, percutaneous coronary intervention (PCI)

## Abstract

**Objective:**

To determine the risk prediction of various degrees of impaired renal function on all‐cause mortality in patients following percutaneous coronary intervention (PCI).

**Background:**

Patients with chronic kidney disease (CKD) are at high risk of all‐cause mortality after PCI. However, there are less data of various degrees of impaired renal function to predict those risks.

**Methods:**

This was a subgroup analysis of nationwide PCI registry of 22 045 patients. Patients were classified into six CKD stages according to preprocedure estimated glomerular filtration rate (eGFR) (ml/min/1.73 m^2^): I (≥90), II (60−89), III (30−59), IV (15−29), or V (<15) without or with dialysis. Baseline clinical and angiographic characteristics were compared among patients in each stage. One‐year all‐cause mortality was reported with risk prediction based on CKD stages and other risk factors.

**Results:**

Patients with CKD stage I−V without and with on dialysis were found in 26.9%, 40.8%, 23.2%, 3.9%, 1.5%, and 3.7%, respectively. PCI procedural success and complication rates ranged from 94.0% to 96.2% and 2.8% to 6.1%, respectively. One‐year overall survival among CKD stages I−V was 96.3%, 93.1%, 84.4%, 65.2%, 68.0%, and 69.4%, respectively (*p *< .001 by log‐rank test). After adjusting covariables, the hazard ratios of all‐cause mortality for CKD stages II−V as compared to stage I by multivariate Cox regression analysis were 1.5, 2.6, 5.3, 5.9, and 7.0, respectively, (*p* < .001).

**Conclusion:**

Among patients undergoing PCI, lower preprocedure eGFR is associated in a dose‐dependent effect with decreased 1‐year survival. This finding may be useful for risk classification and to guide decision‐making.

## 1 INTRODUCTION

Coronary artery disease (CAD) is the leading cause of morbidity and mortality in chronic kidney disease (CKD) patients, accounting for almost half of their deaths.^1^, ^2^ In addition to traditional atherosclerotic risk factors, several uremia‐related risk factors have been associated with accelerated atherosclerosis and aggravated symptoms.^3^, ^4^ Moreover, the survival in those CKD patients is limited by other comorbid diseases that shorten their life‐expectancies.^5^, ^6^, ^7^

There has been controversy regarding how aggressively percutaneous coronary intervention (PCI) should be performed in CKD patients, especially in end‐stage renal disease (ESRD) with dialysis.^8^, ^9^ PCI treatment aims to relieve symptoms, and importantly, to prolong survival. With the advance in new generations of drug‐eluting stents and adjunctive equipment for PCI, the immediate outcomes of PCI have been significantly improved.^10^, ^11^ Nevertheless, previous studies have shown that a lower estimated glomerular filtration rate (eGFR) was associated with decreased long‐term survival.^12^, ^13^ In Thailand, with an increasing burden of CKD, rising PCI costs, and limited healthcare resources, it is necessary to select suitable patients having a better life expectancy.

Although PCI in advanced CKD patients has been studied extensively since the past decade, little is known about the impact of PCI on patients with varying degrees of renal failure. It is well known that CAD and its severity increase as renal function deteriorates.^14^, ^15^ Patients with advanced CKD usually have severe coronary calcification,^16^ extensive CAD disease, and poor LV systolic function, which adversely affects outcomes after revascularization by PCI or even coronary artery bypass grafting (CABG).^17^, ^18^, ^19^ Moreover, PCI in this high‐risk population without renal replacement therapy is associated with a higher risk of contrast‐induced nephropathy and worsening renal function.^20^

In the Asian population, limited published data are existing on quantifying risk and assessment of long‐term outcomes after PCI's treatment in impaired renal function patients, especially with ESRD patients on dialysis. Identifying the poor prognostic factors might help reduce morbidity and mortality, especially in the first year of patients undergoing PCI. Furthermore, if the procedural risk associated with each stage of CKD can be predicted, an operator would be able to weigh the risks and benefits properly, as well as informing PCI decision‐making. Therefore, the objective of this study was to distinguish the risk prediction of various degrees of impaired renal function on all‐cause mortality in patients after undergoing PCI.

## 2 MATERIALS AND METHODS

This study utilized a nationwide prospective multicenter Thai PCI registry, initiated by the Cardiac Intervention Association of Thailand. The registry protocol was published previously^21^ included data from 39 government and private hospitals which they voluntarily participated. All patients enrolled in the study were above 18 years of age and received primary or elective PCI from May 2018 to August 2019. The registry was approved by the Central Research Ethics Committee (COA‐CREC # 006/2018), along with the ethics committee of the Faculty of Medicine, Ramathibodi Hospital, Mahidol University (COA‐MURA2020/974). All patients provided their written informed consent.

Creatinine levels determined before the procedure, along with clinical characteristics and angiographic along with procedural data, were retrieved from the main electronic registry databases. Subject data included the history of cardiovascular risk factors (smoking, hypertension, dyslipidemia, diabetes mellitus [DM], and family history of premature CAD), history of underlying diseases (peripheral arterial disease [PAD], cerebrovascular disease [CVD], and myocardial infarction [MI], heart failure), or prior treatment (PCI and CABG), as well as left ventricular ejection fraction (LVEF).

Angiographic and procedural data included clinical presentation (ST‐elevation myocardial infarction [STEMI], non ST‐elevation myocardial infarction [NSTEMI]/unstable angina [UA], and stable CAD), number of diseased vessels, PCI status (elective, urgent, or emergency), presence of cardiogenic shock, type of contrast and volume, access site, lesion characteristics (lesion complexity, in‐stent restenosis lesion, bypass graft lesion, ostial lesion, and bifurcation lesion), adjunctive devices (rotational atherectomy, cutting/scoring balloon, or laser atherectomy), stent length, and diameter, lesion severity assessment intravascular ultrasound study (IVUS), optical coherence tomography (OCT), or fractional flow reserve wire (FFR)], and intraaortic balloon pump (IABP). Perioperative medications were also recorded along with unfractionated heparin, low‐molecular weight‐heparin (LMWH), glycoprotein IIb/IIIa inhibitor, and P2Y12 inhibitors.

The definition of “angiographic success” was residual stenosis <20% with stent treatment, or <50% with balloon angioplasty alone. Procedural complications were also recorded, including death, MI, stroke, cardiogenic shock, heart failure, a new requirement of dialysis, bleeding required blood transfusion, bleeding within 72 h, endotracheal intubation, cardioversion/defibrillation, and in‐hospital CABG.

### 2.1 Study factor

Impaired renal function was the study factor of interest and was classified into six CKD groups: stage I (GFR ≥ 90), stage II (GFR 60−89), stage III (GFR 30−59), stage IV,^15^, ^16^, ^17^, ^18^, ^19^, ^20^, ^21^, ^22^, ^23^, ^24^, ^25^, ^26^, ^27^, ^28^, ^29^ stage V (GFR < 15 ml/min/1.73 m^2^) without dialysis, and stage V with dialysis. The estimated GFR (ml/min/1.73 m^2^) was calculated based on the CKD epidemiology collaboration (CKD‐EPI) equations.^22^

### 2.2 Outcomes of interest

The primary clinical outcome was 1‐year all‐causes of death. The secondary clinical outcomes were 1‐year fatal and nonfatal MI, fatal and nonfatal stroke, and unplanned revascularization. Death was substantiated by the death certificate, the National Statistics Office, Ministry of Interior. MI was defined as an increase in cardiac troponin (cTn) plus either: (1) evidence of prolonged ischemia as demonstrated by prolonged chest pain (>20 min); or (2) ischemic ST‐segment changes or new pathological Q waves; or (3) angiographic evidence of coronary occlusion or no‐reflow/slow flow; or (4) imaging evidence of new loss of viable myocardium or new regional wall motion abnormality. Stroke was defined as a new neurological deficit during the first 24 h following PCI secondary to cerebral ischemia or cerebral hemorrhage detected by computed tomography or magnetic resonance imaging. Unplanned revascularization was defined as unplanned repeated PCI or CABG. MI during the first 48 h following revascularization was defined as an increase in cTn to >5 × 99th percentile of the upper reference limit (in patients with general baseline cTn concentrations) or an increase of 20% (in patients with elevated cTn before PCI or CABG). All of those adverse outcomes were adjudicated for accuracy by the study committee.

### 2.3 Data collection

Data were initially collected and recorded in the case record forms. Then, it was computerized by well‐trained research assistants. All electronic databases were stored at the Central Data Management Unit, Department of Clinical Epidemiology and Biostatistics, Faculty of Medicine, and Ramathibodi Hospital. Data cleaning and checking were regularly performed after retrieving each survey. In addition, cross‐linked conditions were also developed to ensure the accuracy and consistency of the data.

### 2.4 Statistical analysis

Baseline characteristics were described among CKD groups using means ± SD for continuous variables or as percentages for categorical variables. Continuous data were analyzed using analysis of variance or the Kruskal−Wallis test as appropriate and presented as mean values ± SD. Categorical data were analyzed using the *χ* ^2^ or Fisher's exact test. All tests of significance were two tailed. Hence, Kaplan–Meier overall survival curves were applied to describe survival probabilities by CKD stages I–V with and without dialysis and compared by log‐rank test. Each of the above variables was examined for its association with mortality using a univariate Cox regression analysis. Associated variables with mortality (*p* ≤ .1) in the univariate analysis were also included in the multivariate Cox regression model. The proportionality‐of‐hazards assumption was assessed using the Schoenfeld test, and the assumption was met for all variables included. Adjusted hazard ratios (HR) along with 95% confidence interval (CI) were then estimated.

All analyses were performed using STATA 17.0. (StataCorp. 2021; Stata Statistical Software: Release 17; StataCorp LLC). *p *Value of less than .05 was considered as statistical significance.

## 3 RESULTS

### 3.1 Baseline characteristics of overall data

Out of 22 741 patients, 96.9% (*n* = 22 045) had a baseline eGFR and were included in the analysis. Two‐thirds of patients were in CKD stages I (26.9%) or II (40.8%), while one‐third were in stages III (23.2%), IV (3.9%), or V (without dialysis [1.5%] or with dialysis [3.7%]). Baseline patient characteristics by CKD stages are described in Table 1. In brief, patients with stages III−V were more likely to be older, female, never smokers, and have smaller BMIs and more cardiovascular risk factors (i.e., hypertension, dyslipidemia, and DM). Patients with stage V with dialysis were more likely to have a previous history of CVD, PAD, prior MI, heart failure, PCI, or CABG, and have a lower LVEF.

**Table 1:** Baseline clinical, angiographic and procedural characteristics

Characteristics	Stage I	Stage II	Stage III	Stage IV	Stage V	Stage V	*p* value
					Without dialysis	With dialysis	
	*n* = 5937	*n* = 8990	*n* = 5116	*n* = 860	*n* = 328	*n* = 814	
Age, year, mean (SD)	55.8 ± 9.5	65.2 ± 10.5	70.6 ± 10.4	71.6 ± 10.7	68.2 ± 11.5	65.4 ± 11.1	<.001
Male sex, (%)	74.3	72.6	60.8	50.9	54.3	61.3	<.001
BMI, kg/m^2^, mean (SD)	24.8 ± 4.2	24.3 ± 4.1	24.0 ± 4.2	23.8 ± 4.5	23.4 ± 4.2	23.4 ± 4.4	<.001
Current smoker, (%)	32.6	21.9	17.0	14.4	15.2	6.1	<.001
HT, (%)	55.0	65.9	78.1	83.7	83.5	95.1	<.001
Dyslipidemia, (%)	62.5	66.5	68.3	68.1	62.8	73.0	<.001
DM, (%)	40.1	37.1	53.3	67.7	69.5	71.6	<.001
Cerebrovascular disease, (%)	3.7	5.2	7.6	9.1	7.0	11.4	<.001
Family history of premature CAD, (%)	10.4	9.4	7.5	5.6	7.0	11.1	<.001
PAD, (%)	0.8	1.6	2.2	2.7	1.8	6.5	<.001
Prior MI, (%)	22.9	24.8	25.0	22.0	16.5	23.2	<.001
LVEF, %, mean (SD)	53.7 ± 14.3	52.8 ± 15.5	49.3 ± 16.1	44.3 ± 16.1	47.4 ± 14.5	49.1 ± 14.9	<.001
Prior heart failure, (%)	7.2	10.7	19.6	33.4	25.6	40.8	<.001
Prior PCI, (%)	26.6	31.6	31.3	29.0	22.3	36.7	<.001
Previous CABG, (%)	0.6	1.8	2.3	2.6	0.9	3.6	<.001
CAD presentation, (%)							
STEMI	32.6	25.3	26.0	29.8	26.5	7.0	<.001
NSTEMI/unstable angina	29.4	28.1	31.4	35.3	44.2	40.3	
Stable CAD	38.0	46.6	42.6	34.9	29.3	52.7	
Disease vessel, (%)							
SVD	32.8	26.1	21.5	16.7	21.0	19.2	<.001
DVD	30.6	28.7	27.2	26.6	28.7	26.4	
TVD	(28.0)	(33.2)	(36.8)	(39.7)	(37.2)	(38.6)	
Left main	(8.6)	(11.9)	(14.5)	(17.0)	(13.1)	(15.8)	
Emergency/urgent PCI, (%)	41.2	34.8	38.7	47.7	45.4	25.4	<.001
Cardiogenic shock at start of PCI, (%)	5.0	6.3	11.1	19.4	16.8	5.4	<.001
Volume of contrast, ml, mean (SD)	110.5 ± 54.0	112.3 ± 54.7	108.1 ± 52.9	92.8 ± 46.0	101.2 ± 55.3	109.2 ± 52.4	<.001
Visipaque contrast, (%)	2.6	3.5	9.6	18.1	10.1	4.3	<.001
IABP, (%)	1.4	2.4	5.4	11.0	8.0	3.1	<.001
Femoral access site, (%)	46.0	51.3	57.0	71.2	78.7	84.4	<.001
Unfractionated heparin, (%)	90.1	91.9	91.5	92.6	91.5	93.2	.001
Glycoprotein IIb/IIIa inhibitor, (%)	6.6	5.6	6.5	5.8	4.5	2.5	<.001
Clopidogrel, (%)	92.1	92.0	92.3	94.3	95.7	95.6	<.001

Lesion characteristics	*n* = 7225	*n* = 11 195	*n* = 6413	*n* = 1087	*n* = 410	*n* = 1088	*p* value
Type B2 or C, (%)	75.1	76.8	78.7	78.5	76.8	79.7	<.001
In‐stent restenosis lesion, (%)	5.8	6.1	6.8	6.6	5.9	10.4	<.001
Lesion severity assessment,^a^ (%)	15.8	17.6	16.1	13.6	16.3	21.0	.011
Adjunctive devices,^b^ (%)	3.5	4.6	5.6	4.2	5.9	11.1	<.001
Average stent length per lesion, mm, mean (SD)	24.3 ± 7.8	24.3 ± 7.8	24.0 ± 7.6	24.4 ± 7.9	24.9 ± 8.0	23.7 ± 8.2	.081
Average stent diameter per lesion, mm, mean (SD)	3.0 ± 0.5	3.0 ± 0.5	2.9 ± 0.5	2.9 ± 0.5	2.9 ± 0.5	3.0 ± 0.5	<.001

Abbreviations: BMI, body mass index; CABG, coronary artery bypass surgery; CAD, coronary artery disease; DM, diabetes mellitus; DVD, double vessel disease; HT, hypertension; IABP, intraaortic balloon pump; LVEF, left ventricular ejection fraction; MACE, major adverse cardiac event; MI, myocardial infarction; NSTEMI, non‐ST‐elevation myocardial infarction; PAD, peripheral arterial disease; PCI, percutaneous coronary intervention; SD, standard deviation; STEMI, ST‐elevation myocardial infarction; SVD, single vessel disease; TVD, triple vessel disease.

^a^ Using IVUS, OCT or FFR‐guided.

^b^ Rotational atherectomy, cutting/scoring balloon, or laser atherectomy.

The most common indication for PCI was stable angina, except CKD stages IV and V without dialysis, where NSTEMI/UA was more common. Patients with CKD stage V with dialysis were more likely to present with stable angina and NSTEMI/UA with infrequent STEMI.

### 3.2 Angiographic and procedural characteristics

There were group differences of nearly all lesion characteristics and adjunctive devices used (Table 1). As noted, patients with stage V with dialysis had more lesion complexity (type B2 and C), ostial lesions and in‐stent restenosis, and required IVUS or FFR‐guidance during the procedure or needed plaque modification devices. Patients with stages III−V without dialysis tended to have triple vessels and left main (LM) disease, and to undergo urgent or emergency procedures. Patients in stages IV and V without dialysis had hemodynamic instability with more cardiogenic shock, requiring IABPs. Conversely, for stage V with dialysis, patients were more stable, had planned PCI, and less cardiogenic shock. Femoral access was used more frequently than radial access in stages IV and V patients. Visipaque™ (iodixanol) contrast was usually chosen in stages IV and V without dialysis. Over 90% of patients received unfractionated heparin, but only 10% using received LMWH. Clopidogrel was used more commonly than other new P2Y12 inhibitors (i.e., prasugrel and ticagrelor), especially in the stages IV and V groups. Adjunctive GP IIb/IIIa inhibitor was uncommonly used (<10%) in all groups.

### 3.3 In‐hospital major adverse cardiac event (MACE) in overall population

In‐hospital outcomes of PCI procedures are described in Table 2. The overall angiographic success rates were quite high (approximately 94%−96%) with some differences between groups. The complication rates were similarly high in the patients with stages III, IV, and V without dialysis, with averages of 5%. Interestingly, the lowest complication rate was in the CKD stage V with the dialysis group (2.8%).

**Table 2:** In‐hospital and long‐term outcomes

Characteristics	Stage I	Stage II	Stage III	Stage IV	Stage V	Stage V	*p* value
					Without dialysis	With dialysis	
	*n* = 5937	*n* = 8990	*n* = 5116	*n* = 860	*n* = 328	*n* = 814	
Procedural result							
Angiographic success, (%)	96.2	95.3	94.0	93.6	95.7	94.3	<.001
Procedural complications, (%)	4.8	5.4	6.0	6.1	5.8	2.8	.002
In‐hospital							
All‐cause of death, (%)	0.5	1.6	4.7	11.8	10.1	5.3	<.001
Fatal/nonfatal MI, (%)	5.5	5.3	6.5	8.2	11.0	6.8	<.001
Fatal/nonfatal stroke, (%)	0.3	0.3	0.6	0.7	0.3	0.5	.032
Cardiogenic shock, (%)	4.6	6.3	11.2	20.4	16.8	5.8	<.001
Heart failure, (%)	7.1	10.0	16.2	31.7	29.9	13.9	<.001
New requirement of dialysis, (%)	0.1	0.2	0.7	4.2	5.8	0.0	<.001
Bleeding required blood transfusion, (%)	0.3	0.9	1.6	3.5	2.7	2.1	<.001
Endotracheal intubation, (%)	1.4	3.0	7.2	15.1	12.8	5.4	<.001
Cardioversion/defibrillation, (%)	0.7	1.1	1.9	3.9	2.7	1.7	<.001
In‐hospital CABG, (%)	0.2	0.3	0.5	0.5	0.6	0.5	.123
Length of stay, day, mean (SD)	3.4 ± 7.7	3.5 ± 6.6	4.5 ± 8.2	6.9 ± 13.5	7.6 ± 11.7	6.8 ± 15.0	<.001
At follow‐up 1 year							
All‐cause of death, (%)	3.7	6.9	15.6	34.8	32.0	30.6	<.001
Fatal/nonfatal MI, (%)	6.5	6.2	8.2	9.8	11.9	9.7	<.001
Fatal/nonfatal stroke, (%)	0.7	1.0	1.7	2.1	1.8	1.5	<.001
Unplanned revascularization, (%)	1.9	1.5	1.7	1.7	2.4	3.6	.001

*Note*: Abbreviation as in Table 1.

Overall, in‐hospital all‐causes of death occurred frequently in patients with CKD stages IV and V without dialysis, 11.8%, and 10.1%, respectively. In CKD stages III and V with on dialysis, all causes of death rates were comparable at 4.7% and 5.3%, respectively. The incidence of postprocedural MI, stroke, cardiogenic shock, heart failure, new dialysis requirement, and bleeding events which required blood transfusion were higher in patients with greater CKD stage. The length of hospital stay was longer in the advanced CKD stages.

### 3.4 Long‐term follow‐up results

About 97% of the study patients were followed up to 18 months with a median of 12 ± 3.7 months. All‐cause mortality rates were highest in patients with CKD stages IV and V with/or without dialysis (34.8%, 32.0%, and 30.6%, respectively) followed by stage III, II, and I (15.6%, 6.9%, and 3.7%, respectively) (Table 2), mostly was driven by cardiovascular (CV) death or sudden cardiac death (56.5%). Fatal and nonfatal MI and fatal and nonfatal stroke occurred frequently in patients with CKD stages III−V with or without dialysis. Unplanned revascularization also frequently occurred in patients with stage V without or with dialysis.

### 3.5 Predictors of all‐causes of death

Using a univariate analysis (Table 3), 32 variables were explored concerning mortality. CKD stages, age, female sex, HT, DM, prior MI, prior heart failure, PAD, CAD presentation, disease vessel, PCI status, type B2, or C lesions were significantly associated with increased 1‐year all cause‐mortality. BMI, smoking status, dyslipidemia, prior MI, LVEF, average stent length, and stent diameter per lesion were significantly associated with decreased 1‐year all‐cause mortality. The remaining variables were not significantly related to mortality.

**Table 3:** Describe patient's and lesion's characteristics that associated with 1‐year death: univariate and multivariate

Patient's characteristic	Univariate		Multivariate	
	HR (95% CI)	*p* value	HR (95% CI)	*p* value
CKD				
Stage V with dialysis	9.4 (7.8−11.2)	<.001	7.0 (5.8−8.5)	<.001
Stage V without dialysis	10.1 (8.0−12.8)	<.001	5.9 (4.6−7.5)	<.001
Stage IV	11.5 (9.6−13.7)	<.001	5.3 (4.4−6.4)	<.001
Stage III	4.5 (3.9−5.2)	<.001	2.6 (2.2−3.0)	<.001
Stage II	1.9 (1.6−2.2)	<.001	1.5 (1.2−1.7)	<.001
Stage I	1		1	
Age, year	1.05 (1.05−1.06)	<.001	1.04 (1.03−1.04)	<.001
Female sex	1.5 (1.4−1.6)	<.001		
BMI, kg/m^2^	0.91 (0.90−0.92)	<.001		
Smoking status				
Current smoker	0.89 (0.80−0.98)	.023		
Ex‐smoker	0.72 (0.65−0.79)	<.001		
Never	1			
HT	1.2 (1.1−1.3)	<.001		
DM	1.7 (1.5−1.8)	<.001	1.3 (1.2−1.4)	<.001
Dyslipidemia^a^	0.7 (0.6−0.8)	<.001	0.74 (0.67−0.81)	<.001
Prior MI	0.8 (0.7−0.9)	<.001		
Prior heart failure	2.5 (2.2−2.7)	<.001	1.8 (1.7−2.0)	<.001
LVEF, %	0.960 (0.957−0.963)	<.001		
PAD	2.7 (2.2−3.3)	<.001	2.2 (1.8−2.7)	<.001
CAD presentation				
STEMI	2.7 (2.4−2.9)	<.001	1.9 (1.8−2.2)	.002
NSTEMI/unstable angina	1.7 (1.5−1.9)	<.001	1.1 (1.0−1.3)	.051
Stable CAD	1		1	
Disease vessel				
Left main	1.92 (1.69−2.19)	<.001	1.6 (1.4−1.7)	<.001
TVD	1.19 (1.06−1.33)	.003		
DVD	1.08 (0.96−1.22)	.201		
SVD	1			
PCI status				
Emergency	3.3 (3.0−3.6)	<.001	1.9 (1.7−2.2)	<.001
Urgent	1.7 (1.6−2.0)	<.001		
Elective	1			
Type B2 or C lesions	1.4 (1.3−1.6)	<.001	1.3 (1.2−1.5)	<0.001
Average stent length per lesion	0.992 (0.986−0.997)	.004		
Average stent diameter per lesion	0.68 (0.63−0.75)	<.001		

Abbreviation: CI, confidence interval.

*Note*: Abbreviation as in Table 1.

^a^ Dyslipidemia treated with statin.

Therefore, all these variables were used in a multivariable Cox regression model. CKD stages, age, DM, prior heart failure, PAD, STEMI, LM, emergent/urgent PCI status, and type B2 or C lesions shown to be independently related to mortality (Table 3). As noted, dyslipidemia was the only risk reduction of mortality.

**Figure 1:**
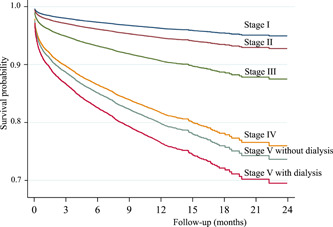
Kaplan−Meier survival for 1‐year mortality by CKD stages. CKD, chronic kidney disease.

One‐year overall survival among CKD stages I–V without and with on dialysis was 96.3%, 93.1%, 84.4%, 65.2%, 68.0%, and 69.4%, respectively (*p* < .001 by log‐rank test). After adjusting for covariables, CKD stages were still a strong predictor of 1‐year all‐cause mortality. The HR of all‐cause mortality for CKD stages II–V compared to stage 1 by multivariate Cox regression analysis were as follows: 1.5, 2.6, 5.3, 5.9, and 7.0, respectively (*p* < .001). Kaplan−Meier curves were used to describe survival probabilities by CKD groups (Figure 1). The differences between CKD stages I−III and stages IV−V without and with dialysis were visible as early as the first month, and persisted through the follow‐up period.

## 4 DISCUSSION

Using the large national PCI registry, it was found that impaired renal function measured by lower preprocedure eGFR is associated with a dose‐dependent effect with decreased 1‐year survival in patients following successful PCI. This finding may be useful for identifying patients at risk and guiding decision‐making. Other eight variables (old age, DM, prior heart failure, PAD, STEMI, LM lesion, emergent/urgent PCI status, and type B2 or C lesions) were well‐known risk predictors and independently associated with all‐cause mortality. Only dyslipidemia was a risk reduction and probably caused by the statin treatment effect.

Based on our contemporary PCI experience, the consecutively registered patients with a variety of clinical presentations from stable CAD to MI were stratified by baseline eGFR into six groups. There was a high prevalence of patients with mildly impaired (eGFR 60−90) or overtly impaired (eGFR < 60) renal function; only one‐quarter of patients had normal renal function (eGFR > 90). Subjects with CKD stage II–V without dialysis were found to be at strong risk for in‐hospital and 1‐year mortality after successful PCI. This dose‐dependent lower GFR associated with decreased long‐term survival is similar to the previous study by Patel et. al.^23^ The 5‐year Kaplan–Meier overall survival among CKD stages I–V was 98.1%, 95.5%, 91.8%, 82.5%, and 76.9%, respectively. This study's survival rates were quite comparable but with a shorter follow‐up period. The difference is that this study also focused on stage V with dialysis which revealed a 69.4% of 1‐year survival.

As known, patients with CKD stage V with regular dialysis are at high risk for CV mortality and morbidity.^24^, ^25^ They have been largely excluded from all randomized trials evaluating outcomes of revascularization. In general, PCI in these advanced CKD patients, especially stage V on dialysis, is more complex and difficult to perform because of greater calcification and lesion complexity, requiring plaque modification devices such as atherectomy.^26^ However, our findings showed that the angiographic success rate was acceptably as high as other CKD stages whereas the complication rate was unexpectedly the lowest in CKD with dialysis than the other groups. There are several possible explanations for these findings, including younger age patients, less STEMI presentation, less cardiogenic shock and mostly of the PCI procedure were planned. Our study supported the previous studies that contemporary PCI is feasible and safe in appropriated selected patients with higher technical success and acceptable complication rates, even though they require more complex PCI procedure.^27^ However, it needs to be emphasize that the 1‐year all‐cause mortality in these patients was still high after adjustment for other risk factors which mostly was driven by CV death or sudden death (56.5%) and additional unplanned revascularization.

Antecedently, there was no single eGFR cutoff value that was a strong predictor of in‐hospital and long‐term mortality after PCI. Recently, Nozari, et al.^28^ found that a eGFR < 30 ml/min/1.73 m^2^ is a strong predictor of 5‐year MACEs (cardiac death, MI, CABG, rehospitalization due to UA, and revascularization) and suggested that PCI is more suitable and safer in patients with eGFRs > 30 ml/min/1.73 m^2^. However, there is also evidence that even mild renal impairment (eGFR 60−90) is associated with an increase in the incidence of in‐hospital MACEs after PCI.^17^, ^29^, ^30^ To assess these risks before PCI, it is important to encourage that all patients should have a baseline renal function measured by eGFR determined, not just a creatinine level. This value more accurately reflects renal function and is more relevant to individual risk levels.

In general, the decision of whether a patient should be treated with medical treatment or undergo revascularization (either PCI or CABG) depended on the patient's condition and the operator's judgment. In CKD patients (eGFR < 60 ml/min/1.73 m^2^) with stable CAD, PCI did not reduce the risk of death or MI when added to optimal medical treatment.^31^ Similarly to the ISCHEMIA‐CKD trial in advanced CKD (eGFR < 30 ml/min/1.73 m^2^) with stable CAD and moderate to severe ischemia on noninvasive stress testing, initial invasive strategy with PCI, as compared with an initial conservative strategy, failed to reduce the risk of death or nonfatal MI.^32^

A majority of the study patients with advanced CKD presented with NSTEMI/UA or STEMI, and required that PCI be done on an urgent or emergency basis. These patients usually present with more comorbidities, more complex lesions, and hemodynamic instability when compared with the normal or mild impaired renal function population. As noted, CKD patients with STEMI receive significantly less PCI compared with patients without CKD. A previous study has shown that CKD was associated with adverse outcomes in patients presenting with STEMI and reduced the likelihood of successful fibrinolysis and increased the risk of short‐term MACEs.^33^ On the opposite, coronary revascularization for STEMI in CKD patients was associated with lower mortality compared to medical management.^34^, ^35^

Unlike STEMI, the role of PCI compared with medical treatment in patients with NSTEMI and advanced CKD is still uncertain, given the increased risk of procedural complications. Recently, using the largest, in‐hospital database in the United States, Bhatia, et al.^36^ reported the decreased use of PCI among NSTEMI patients as CKD severity increases and the greater of all‐causes, in‐hospital mortality in NSTEMI patients with more severe CKD regardless of the treatment strategies. Nevertheless, the author confirmed that patients with CKD presenting with NSTEMI appear to benefit from PCI compared with medical therapy. Hence, these findings concurred with several previous studies that demonstrated the efficacy and safety of invasive management with PCI in CKD patients with NSTEMI compared with medical management.^37^, ^38^, ^39^

In summary, the severity of renal function impairment, comorbid diseases, clinical presentation, and coronary complexity and severity are the major component for making a decision making. From the results of previous studies, it seems that CKD patients should not be deprived of standard treatment but how invasive management ought to be titrated to avoid adverse outcomes is a major issue to consider. Using this study's findings, patients with eGFR >30 ml/min/1.73 m^2^ would be a suitable cutoff for using to consider early invasive management. Importantly, only culprit‐PCI may be a better reperfusion option for CKD patients with STEMI or NSTEMI with the multivessel disease rather than multivessel‐PCI, concerning the procedure time and the risk of contrast‐induced nephropathy. To answer these uncertainties, further well‐designed, large‐scale prospective studies, and randomized controlled trials are warranted to substantiate these findings and to assess the best revascularization strategies in each sub‐group of this high‐risk population.

### 4.1 Limitation of the study

Though this was a well‐designed, and prospective cohort study, some limitations need to be mentioned. First, this was only one eGFR result from before the procedure to use for stratification of patients. The subsequent effect on renal function, whether it deteriorated or improved, was not known. Second, we assessed direct effect of CKD on mortality but CKD might be a surrogate marker for other comorbidities that are also causes of mortality. To prove this, a mediation analysis should be further applied. Third, some data were incomplete and not included for analysis such as a syntax score, bleeding risk, which can be important for decision making. Forth, the efficacy of the treatment in each group could not be evaluated because of the lack of randomization and compliance of treatment. Fifth, the different treatment effect and prognoses of patients in each CKD group were not compared because these were out of scopes of this study and they depended on various uncontrolled factors, including clinical profile and anatomical differences. A randomized controlled trial or a real‐world data with propensity score analysis comparing long‐term survival after different treatment modalities in these CKD stages should be further conducted.

## 5 CONCLUSION

CKD is a common comorbid disease in CAD patients who undergo elective or urgent/emergency PCI. Various stages of CKD are independently associated with 1‐year mortality even after adjustment for other risk factors. Deterioration of renal function, even to a mild degree, was a strong independent risk predictor of in‐hospital MACEs and 1‐year all‐cause mortality in a dose‐dependent effect after successful PCI. Currently, the choice of coronary revascularization in these high‐risk patients is individualized. This finding may be advantageous for risk classification and to guide decision‐making. Whether this risk prediction of the CKD stage can help to reduce the long‐term all‐cause mortality is a question requiring further study.

## CONFLICT OF INTEREST

The authors declare no conflict of interest.

## Data Availability

The data that support the findings of this study are available from the corresponding author upon reasonable request.

## References

[1] Collins AJ , Foley RN , Herzog C , et al. US renal data system 2012 annual data report. Am J Kidney Dis. 2013;61(suppl 1):A7 , e1‐476. 23253259 10.1053/j.ajkd.2012.11.031

[2] Herzog CA , Ma JZ , Collins AJ . Poor long‐term survival after acute myocardial infarction among patients on long‐term dialysis. N Engl J Med. 1998;339:799‐805. 9738087 10.1056/NEJM199809173391203

[3] Muntner P , He J , Astor BC , Folsom AR , Coresh J . Traditional and nontraditional risk factors predict coronary heart disease in chronic kidney disease: results from the atherosclerosis risk in communities study. J Am Soc Nephrol. 2005;16:529‐538. 15625072 10.1681/ASN.2004080656

[4] Sarnak MJ , Amann K , Bangalore S , et al. Chronic kidney disease and coronary artery disease: JACC state‐of‐the‐art review. J Am Coll Cardiol. 2019;74:1823‐1838. 31582143 10.1016/j.jacc.2019.08.1017

[5] Enríquez J , Bastidas M , Mosquera M , et al. Survival on chronic dialysis: 10 years' experience of a single Colombian center. Adv Perit Dial. 2005;21:164‐167. 16686311

[6] Damman K , Valente MAE , Voors AA , O'Connor CM , van Veldhuisen DJ , Hillege HL . Renal impairment, worsening renal function, and outcome in patients with heart failure: an updated meta‐analysis. Eur Heart J. 2014;35:455‐469. 24164864 10.1093/eurheartj/eht386

[7] Chantrel F , de Cornelissen F , Deloumeaux J , Lange C , Lassalle M . Registre REIN. [survival and mortality in ESRD patients]. Nephrol Ther. 2013;9(suppl 1):S127‐S137. 24119578 10.1016/S1769-7255(13)70042-7

[8] Rubenstein MH , Harrell LC , Sheynberg BV , Schunkert H , Bazari H , Palacios IF . Are patients with renal failure good candidates for percutaneous coronary revascularization in the new device era? Circulation. 2000;102:2966‐2972. 11113047 10.1161/01.cir.102.24.2966

[9] Scholz SS , Lauder L , Ewen S , et al. One‐year clinical outcomes in patients with renal insufficiency after contemporary PCI: data from a multicenter registry. Clin Res Cardiol. 2020;109:845‐856. 31792571 10.1007/s00392-019-01575-y PMC7308257

[10] Holzmann MJ , Siddiqui AJ . Outcome of percutaneous coronary intervention during non‐ST‐segment‐elevation myocardial infarction in elderly patients with chronic kidney disease. J Am Heart Assoc. 2020;9:e015084. 32519559 10.1161/JAHA.119.015084 PMC7429052

[11] Kim S‐M , Tripathy DR , Park SW , et al. Impact of chronic kidney disease on clinical outcomes in diabetic patients undergoing percutaneous coronary intervention in the era of newer‐generation drug‐eluting stents. Korean Circ J. 2017;47:222‐230. 28382078 10.4070/kcj.2016.0312 PMC5378029

[12] Papachristidis A , Lim WY , Voukalis C , Ayis S , Laing C , Rakhit RD . Determinants of mortality in patients with chronic kidney disease undergoing percutaneous coronary intervention. Cardiorenal Med. 2016;6:169‐179. 27275153 10.1159/000442897 PMC4886034

[13] Patel AD , Ibrahim M , Swaminathan RV , et al. Five‐year mortality outcomes in patients with chronic kidney disease undergoing percutaneous coronary intervention. Catheter Cardiovasc Interv. 2017;89:E124‐E132. 27519355 10.1002/ccd.26664

[14] Chronic Kidney Disease Prognosis Consortium , Matsushita K , van der Velde M , Astor BC , et al. Association of estimated glomerular filtration rate and albuminuria with all‐cause and cardiovascular mortality in general population cohorts: a collaborative meta‐analysis. Lancet. 2010;375:2073‐2081. 20483451 10.1016/S0140-6736(10)60674-5 PMC3993088

[15] Manjunath G , Tighiouart H , Ibrahim H , et al. Level of kidney function as a risk factor for atherosclerotic cardiovascular outcomes in the community. J Am Coll Cardiol. 2003;41:47‐55. 12570944 10.1016/s0735-1097(02)02663-3

[16] Moe SM , Chen NX . Mechanisms of vascular calcification in chronic kidney disease. J Am Soc Nephrol. 2008;19:213‐216. 18094365 10.1681/ASN.2007080854

[17] Best PJ , Lennon R , Ting HH , et al. The impact of renal insufficiency on clinical outcomes in patients undergoing percutaneous coronary interventions. J Am Coll Cardiol. 2002;39:1113‐1119. 11923033 10.1016/s0735-1097(02)01745-x

[18] Cai Q , Mukku VKAM . Coronary artery disease in patients with chronic kidney disease: a clinical update. Curr Cardiol Rev. 2013;9:331‐339. 24527682 10.2174/1573403X10666140214122234 PMC3941098

[19] Tadros GM , Herzog CA . Percutaneous coronary intervention in chronic kidney disease patients. J Nephrol. 2004;17:364‐368. 15365955

[20] Rear R , Bell RM , Hausenloy DJ . Contrast‐induced nephropathy following angiography and cardiac interventions. Heart. 2016;102:638‐648. 26857214 10.1136/heartjnl-2014-306962 PMC4819627

[21] Sansanayudh N , Srimahachota S , Chandavimol M , Limpijankit T , Kehasukcharoen W . Multi‐center, prospective, nation‐wide coronary angioplasty registry in Thailand (Thai PCI registry): registry design and rationale. J Med Assoc Thai. 2021;104:1678‐1685.

[22] Levey AS , Stevens LA , Schmid CH , et al. A new equation to estimate glomerular filtration rate. Ann Intern Med. 2009;150:604‐612. 19414839 10.7326/0003-4819-150-9-200905050-00006 PMC2763564

[23] Patel B , Shah M , Dusaj R , Maynard S , Patel N . Percutaneous coronary intervention and inpatient mortality in patients with advanced chronic kidney disease presenting with acute coronary syndrome. Baylor Univ Med Cent Proc. 2018;30:400‐403. 10.1080/08998280.2017.11930205 PMC5595374 28966444

[24] de Lemos JA , Hillis LD . Diagnosis and management of coronary artery disease in patients with end‐stage renal disease on hemodialysis. J Am Soc Nephrol. 1996;7:2044‐2054. 8915964 10.1681/ASN.V7102044

[25] Naidu SS , Selzer F , Jacobs A , et al. Renal insufficiency is an independent predictor of mortality after percutaneous coronary intervention. Am J Cardiol. 2003;92:1160‐1164. 14609589 10.1016/j.amjcard.2003.07.023

[26] Schwarz U , Buzello M , Ritz E , et al. Morphology of coronary atherosclerotic lesions in patients with end‐stage renal failure. Nephrol Dial Transplant. 2000;15:218‐223. 10648668 10.1093/ndt/15.2.218

[27] Nakachi T , Kohsaka S , Yamane M , et al. Impact of hemodialysis on procedural outcomes of percutaneous coronary intervention for chronic total occlusion: insights from the Japanese multicenter registry. J Am Heart Assoc. 2017;6:e006431. 29021271 10.1161/JAHA.117.006431 PMC5721853

[28] Nozari Y , Shafiee A , Kassaian SE , Jalali A , Roozbeh M , Safarian H . Effect of various degrees of chronic kidney disease on long‐term outcome of patients with percutaneous coronary intervention. Arch Iran Med. 2019;22:247‐251. 31256597

[29] Lopes NH , da Silva Paulitsch F , Pereira A , et al. Mild chronic kidney dysfunction and treatment strategies for stable coronary artery disease. J Thorac Cardiovasc Surg. 2009;137:1443‐1449. 19464462 10.1016/j.jtcvs.2008.11.028

[30] Grandjean‐Thomsen NLo , Marley P , Shadbolt B , Farshid A . Impact of mild‐to‐moderate chronic kidney disease on one year outcomes after percutaneous coronary intervention. Nephron. 2017;137:23‐28. 28478459 10.1159/000473863

[31] Sedlis SP , Jurkovitz CT , Hartigan PM , et al. Optimal medical therapy with or without percutaneous coronary intervention for patients with stable coronary artery disease and chronic kidney disease. Am J Cardiol. 2009;104:1647‐1653. 19962469 10.1016/j.amjcard.2009.07.043

[32] Bangalore S , Maron DJ , O'brien SM , et al. Management of coronary disease in patients with advanced kidney disease. N Engl J Med. 2020;382:1608‐1618. 32227756 10.1056/NEJMoa1915925 PMC7274537

[33] Xie W , Patel A , Boersma E , et al. Chronic kidney disease and the outcomes of fibrinolysis for ST‐segment elevation myocardial infarction: a real‐world study. PLoS One. 2021;16:e0245576. 33465135 10.1371/journal.pone.0245576 PMC7815111

[34] Panchal HB , Zheng S , Devani K , et al. Impact of chronic kidney disease on revascularization and outcomes in patients with ST‐elevation myocardial infarction. Am J Cardiol. 2021;150:15‐23. 34006375 10.1016/j.amjcard.2021.03.057

[35] Kawsara A , Sulaiman S , Mohamed M , et al. Treatment effect of percutaneous coronary intervention in dialysis patients with ST‐elevation myocardial infarction. Am J Kidney Dis. 2021;79:P832‐P840. 10.1053/j.ajkd.2021.08.023 34662690

[36] Bhatia S , Arora S , Bhatia SM , et al. Non‐ST‐segment‐elevation myocardial infarction among patients with chronic kidney disease: a propensity score‐matched comparison of percutaneous coronary intervention versus conservative management. J Am Heart Assoc. 2018;7:1‐9. 10.1161/JAHA.117.007920 PMC5907556 29525779

[37] Ankur K , Monil M , Gabriel I , et al. Invasive versus medical management in patients with chronic kidney disease and non‐ST‐elevation myocardial infarction. J Am Coll Cardiol. 2021;78(suppl 19):B7.

[38] Kim YH , Her AY , Jeong MH , et al. Outcome of early versus delayed invasive strategy in patients with non‐ST‐segment elevation myocardial infarction and chronic kidney disease not on dialysis. Atherosclerosis. 2022;344:60‐70. 34924173 10.1016/j.atherosclerosis.2021.11.024

[39] Franczyk‐Skóra B , Gluba A , Banach M , Rysz J . Treatment of non‐ST‐elevation myocardial infarction and ST‐elevation myocardial infarction in patients with chronic kidney disease. Arch Med Sci. 2013;6:1019‐1027. 10.5114/aoms.2013.39792 PMC3902722 24482645

